# Soloxolone methyl inhibits influenza virus replication and reduces virus-induced lung inflammation

**DOI:** 10.1038/s41598-017-14029-0

**Published:** 2017-10-25

**Authors:** Andrey V. Markov, Alexandra V. Sen’kova, Dawid Warszycki, Oksana V. Salomatina, Nariman F. Salakhutdinov, Marina A. Zenkova, Evgeniya B. Logashenko

**Affiliations:** 1 Institute of Chemical Biology and Fundamental Medicine, Siberian Branch Russian Academy of Sciences, 8, Lavrent’ev ave., Novosibirsk, 630090 Russian Federation; 2 Institute of Pharmacology, Polish Academy of Sciences, 12, Smętna street, Kraków, 31-343 Poland; 3 N. N. Vorozhtsov Novosibirsk Institute of Organic Chemistry, Siberian Branch Russian Academy of Sciences, 9, Lavrent’ev ave., Novosibirsk, 630090 Russian Federation

## Abstract

Highly pathogenic influenza viruses pose a serious public health threat to humans. Although vaccines are available, new antivirals are needed to efficiently control disease progression and virus transmission due to the emergence of drug-resistant viral strains. In this study, we describe the anti-viral properties of Soloxolone methyl (SM) (methyl 2-cyano-3,12-dioxo-18βH-olean-9(11),1(2)-dien-30-oate, a chemical derivative of glycyrrhetinic acid) against the flu virus. Anti-flu efficacy studies revealed that SM exhibits antiviral activity against the H1N1 influenza A virus in a dose-dependent manner causing a more than 10-fold decrease in virus titer and a reduction in the expression of NP and M2 viral proteins. In a time-of-addition study, SM was found to act at an early stage of infection to exhibit an inhibitory effect on both the attachment step and virus uptake into cells. Also, in infected cells SM downregulates the expression of the inflammatory cytokines IL-6 and TNF-α. In infected mice, SM administered intranasally prior to and after infection significantly decreases virus titers in the lung and prevents post-challenge pneumonia. Together, these results suggest that Soloxolone methyl might serve as an effective therapeutic agent to manage influenza outbreaks and virus-associated complications, and further preclinical and clinical investigation may be warranted.

## Introduction

Viral respiratory infections are the most common diseases experienced by people of all ages. Influenza A virus (IVA) is considered to be a major human pathogen and can cause between 3 and 5 million cases of severe illness in a normal season and up to 500,000 deaths worldwide^1^. Due to the emergence of new pandemic strains through viral mutation and reassortment, as exemplified by the 2009 H1N1 influenza pandemic^2^, the IVA causes acute respiratory infections in humans, with severities ranging from morbidity to mortality. National Influenza Centers (NICs) and other national influenza laboratories in 96 countries, areas or territories have reported data for the time period from 22 February 2016 to 06 March 2016. Among 159,429 tested specimens during that time period, 47,202 was shown to be positive for influenza viruses, of which 35,026 (74.2%) were typed as influenza A. Of the sub-typed influenza A viruses, 15,851 (87.3%) were influenza A (H1N1) pdm09 (WHO, 2016). Annual outbreaks of IVA in recent years (2010–2013) in the United States alone claimed over 45,000 lives^3^ and cost billions of dollars. There is concern that the 2009 H1N1 virus will continue to cause serious disease in the immediate future^4^. Although annual vaccination is the primary strategy for the prevention of infections, influenza antiviral drugs play an important role in the comprehensive approach to the control of illness and transmission.

Currently, there are only two classes of US FDA-approved antiviral drugs available for the treatment and prevention of influenza: inhibitors of M2 ion channels (the adamantane derivatives amantadine and rimantadine) and neuraminidase inhibitors (NAIs; zanamivir, oseltamivir and peramivir)^5^. In addition, in a handful of countries, clinical use has been approved for favipiravir (Japan) – an inhibitor of RNA-dependent RNA polymerase^4^ and the hemagglutinin inhibitor arbidol (Russia, China)^6^, but their use is highly restricted. Besides these four groups of anti-influenza drugs, there are several other approaches under investigation, including blockers of viral ribonucleoprotein complex (vRNPs) formation, inhibitors of NS1 function^7^, inhibitors of virus attachment, endocytosis and fusion^5^, and oligonucleotide-based antivirals^8^, but so far no alternative drugs have been licensed.

The targets of most types of drugs are viral proteins, and for optimum efficacy they must be administered within 48 h of symptom onset. The adamantanes are specific for influenza A virus, and act by inhibiting M2 ion channel activity by blocking the migration of H^+^ ions into the interior of virus particles within endosomes, which is a process that is needed for uncoating to occur^9^. However, their wide use has been limited due to the rapid emergence of drug resistance, the ready transmissibility of drug-resistant viruses and the occurrence of central nervous system side effects^10^.

The current standard-of-care antivirals for influenza cases are potent inhibitors of influenza virus neuraminidase (NA) – a surface glycoprotein anchored in the viral envelope with sialidase activity critical for the release of progeny virions from infected cells^6^. NAIs therefore prevent the infection of new host cells and, as a result, halt the spread of infection in the respiratory tract. While these agents possess high affinity and specificity for a variety of influenza viruses, they suffer from limitations in their efficacy due to adverse effects and drug resistance^11^. For oseltamivir, drug-resistant strains have increasingly emerged since 2007. There were many more reported cases of oseltamivir-resistant influenza A (H1N1) pdm09 infections with the H275Y NA mutation during 2011 than during the first year of the pandemic^12^. The appearance of drug-resistant influenza viruses is caused either by a mutation in the active site of the NA, which alters its sensitivity to inhibitors^13^, or a mutation in influenza virus hemagglutinin (HA), thus decreasing the affinity of HA for cellular receptors and therefore allowing efficient release of progeny virions from infected cells almost regardless of NA activation status^13^. These reasons limit the future utility of commonly used NA inhibitors, highlighting the urgent need for new classes of anti-influenza agents to combat potential human influenza pandemics.

Plants represent important sources of lead compounds, with up to 40% of modern drugs being derived from plant materials^14^. The search for plant-based antivirals against the influenza virus is promising, as several plants have been shown to possess anti-influenza activity.

Licorice root has been used since ancient Egyptian, Greek, and Roman times in the West and in ancient China, Tibet and India in the East. One of the main bioactive components of licorice root is glycyrrhetinic acid (GLA), which possesses a great variety of pharmacological and biological properties^15–17^.

**Figure 1:**
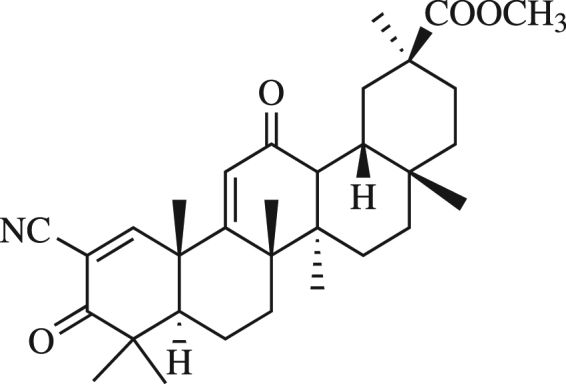
Chemical structure of methyl 2-cyano-3,12-dioxo-18βH-olean-9(11),1(2)-dien-30-oate (Soloxolone methyl, SM).

Earlier, we reported that Soloxolone methyl (methyl 2-cyano-3,12-dioxo-18βH-olean-9(11),1(2)-dien-30-oate, or SM) a semisynthetic derivative, obtained by direct modification of the A- and C-rings of GLA (Fig. 1), possesses high antiproliferative and pro-apoptotic activities with respect to different cancer cell lines^18,19^. Also, we showed that SM displays anti-inflammatory activity. SM causes a significant and dose-dependent decrease in NO production in LPS activated J774 murine macrophages^20^.

In this study, we analyzed the anti-influenza activity of SM *in vitro* and *in vivo*. Different SM treatment protocols in synchronized infections were applied to elucidate the phase of the viral reproductive cycle that is affected by SM. Moreover, we investigated the effects of SM on influenza A pneumonia in mice and reveal the potential of SM in the prevention of influenza infection.

## Results

### SM inhibits the growth of H1N1 influenza A in MDCK and A549 cells

**Figure 2:**
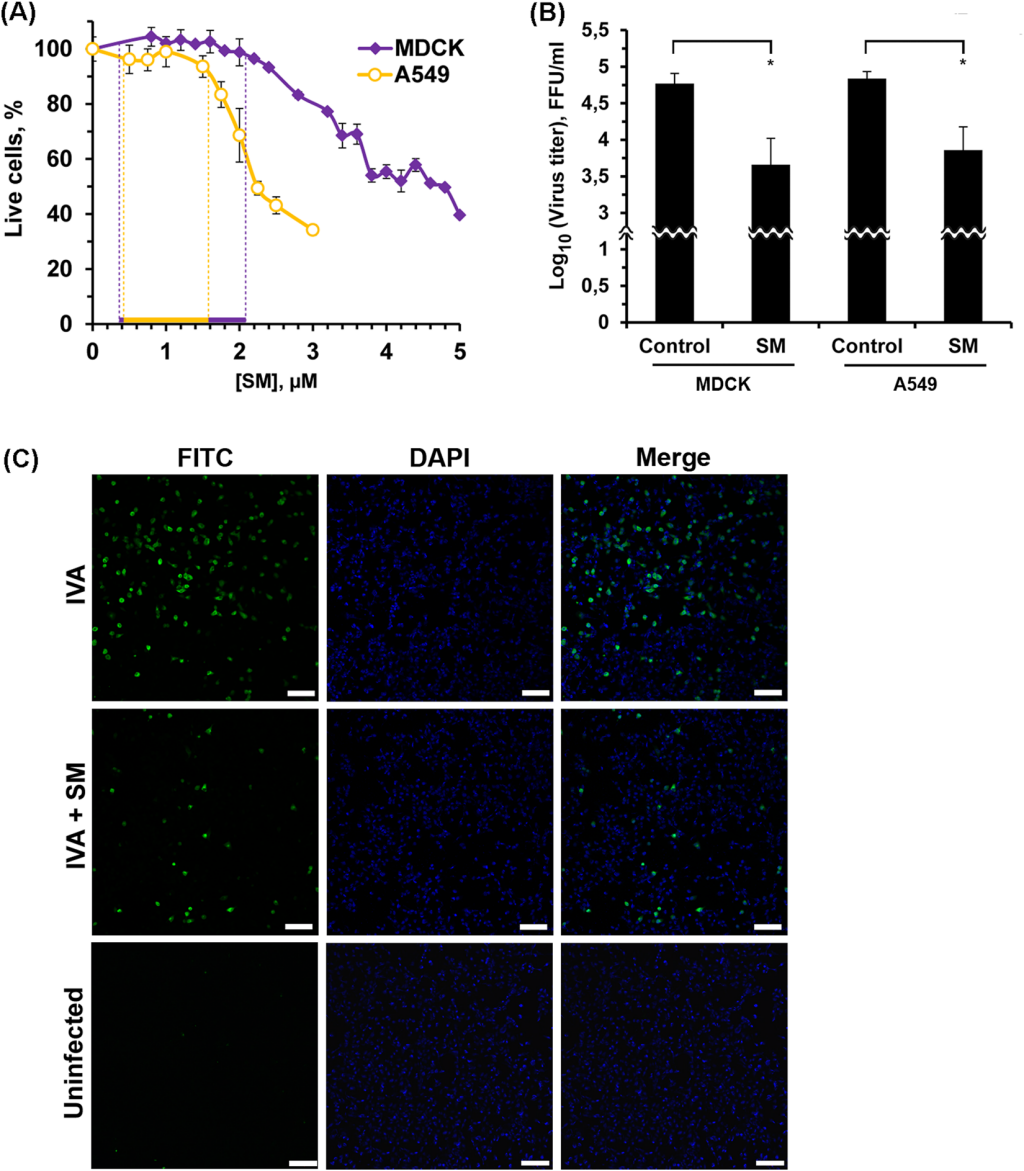
SM reduces IVA replication. (**A**) Dose response curves of SM for MDCK and A549 cell lines. Cells were incubated with increasing concentrations of SM for 24 h. After incubation, cell viability was measured by MTT assay. The results are expressed as a percentage of viable cells observed after treatment with compounds vs control cells (100%) incubated in the presence of 0.1% DMSO. The data are obtained from at least three independent experiments done in triplicate. Dashed line indicates the nontoxic span of SM concentrations. (**B**) Effect of SM on IVA reproduction on different epithelial cell lines. MDCK and A549 cells were infected with IVA at MOI 0.01. After 1 h adsorption at standard conditions, the monolayers were washed twice with PBS and incubated at standard conditions in fresh infection medium with SM (1 µM) for 24 h. Viral yields in supernatants were determined by FFA. Control – untreated IVA-infected cells. SM – SM-treated IVA-infected cells. Error bars represent the standard deviation of three independent experiments performed in triplicate. P value: *<0.05. **(C)** Effects of SM on the expression of IVA nucleoprotein (NP) in A549 cells. Confluent A549 cells were infected with IVA (MOI = 0.1) and then treated with SM (1 µM). At 6 h p.i. the cells were fixed with paraformaldehyde and expression of viral NP protein was detected by IFA (FITC corresponding to NP protein, nuclei were counterstained with DAPI). Cells were viewed using laser scanning confocal microscope with a 10× objective. Bars, 100 µm.

Since previous studies have shown SM cytotoxicity in cancer cell line^18,19^ a viability assay based on MTT was performed in the present study to determine the non-specific cytotoxicity of SM for MDCK and A549 cells. From the data on MDCK and A549 cell viability (Fig. 2A) it is clearly seen that SM was not toxic for cells in concentrations up to 2 µM and 1.5 µM for MDCK and A549 cells, respectively. Therefore, this span of concentrations was used for the subsequent experiments in this study.

MDCK is noted for its highly efficient replication of the IVA^21–23^ and is used routinely in plaque and focus forming assays. To examine the influence of SM on virus progeny, MDCK cells were infected with IVA at MOI 0.01 and infectious virus titers were determined 24 h post infection with FFA (Fig. 2B). It was shown, that the incubation of infected cells with 1 µM SM caused pronounced antiviral effects (10-fold in the virus titer). Observed reduction of IVA titer was dose-dependent (Supplementary Fig. S1) and was not because of the non-specific cytotoxicity of SM for MDCK cells. We showed that SM does not decrease viability of infected cells in the nontoxic concentration span even applied simultaneously with IVA (Supplementary Fig. S1). Since epithelial cells are a key host target cells for influenza A *in vivo*, the effect of SM was also tested on A549 human lung epithelial cells (derived from a human pulmonary adenocarcinoma), which are an established model for type II pneumocytes^24^. We showed that SM inhibited the production of virus progeny in A549 cells, similar to observations in MDCK cells (Fig. 2B).

We used glycyrrhizin (GLZ) – parent compound for SM (an aglycone of GLZ, glycyrrhetinic acid, was used as the starting material for SM synthesis) as a control since earlier this triterpenoid was shown to display anti-influenza activity. We found that GLZ does not affect virus progeny even at 1 mM that exceed the concentration of SM by three orders of magnitude (Supplementary Table S1). Probably GLZ activity against IVA depends on the virus strain (a decrease in log_10_ (virus titer) by 1 unit was observed from 0.2 mM to 1 mM GLZ for H5N2^25^ and H3N2^26^, respectively) (Supplementary Table S1).

The effect of SM on virus progeny was supported by assessing the expression of viral NP protein by an indirect immunofluorescence assay. As shown in Fig. 2C, treatment of infected cells with SM caused a significant decrease of NP virus protein signal. Similar results were obtained for M2 protein (Supplementary Fig. S2).

### Studies on the mechanism of viral growth inhibition by SM

First, we evaluated whether SM would be capable of neutralizing virus HA, resulting in the inhibition of binding of this viral surface protein to its receptors. Influenza virus HA mediates the attachment of virions to sialic acid residues found on the glycoproteins and glycolipids of host cells, which is a critical step in the initiation of infection^27^. Similarly, viral HA binds to sialic acids expressed on the surface of erythrocytes, resulting in hemagglutination. Thus, we examined the ability of SM to inhibit virus-induced hemagglutination using a hemagglutination assay. It was found that SM at concentrations from 0.1 to 100 µM did not inhibit virus binding to its receptor (Supplementary Fig. S3).

The influenza virus NA glycoprotein has sialidase activity and mediates the release of viral progeny from infected cells, thus promoting virus transmission and spread^28^. In addition, viral NA removes sialic acid from glycans expressed by the viral HA glycoprotein, thereby preventing self-aggregation of virions^29^. SM was tested for its inhibitory activity against influenza virus NA using the NA-Fluor^TM^ Influenza Neuraminidase Assay Kit, but no effect was observed (Supplementary Fig. S4).

**Figure 3:**
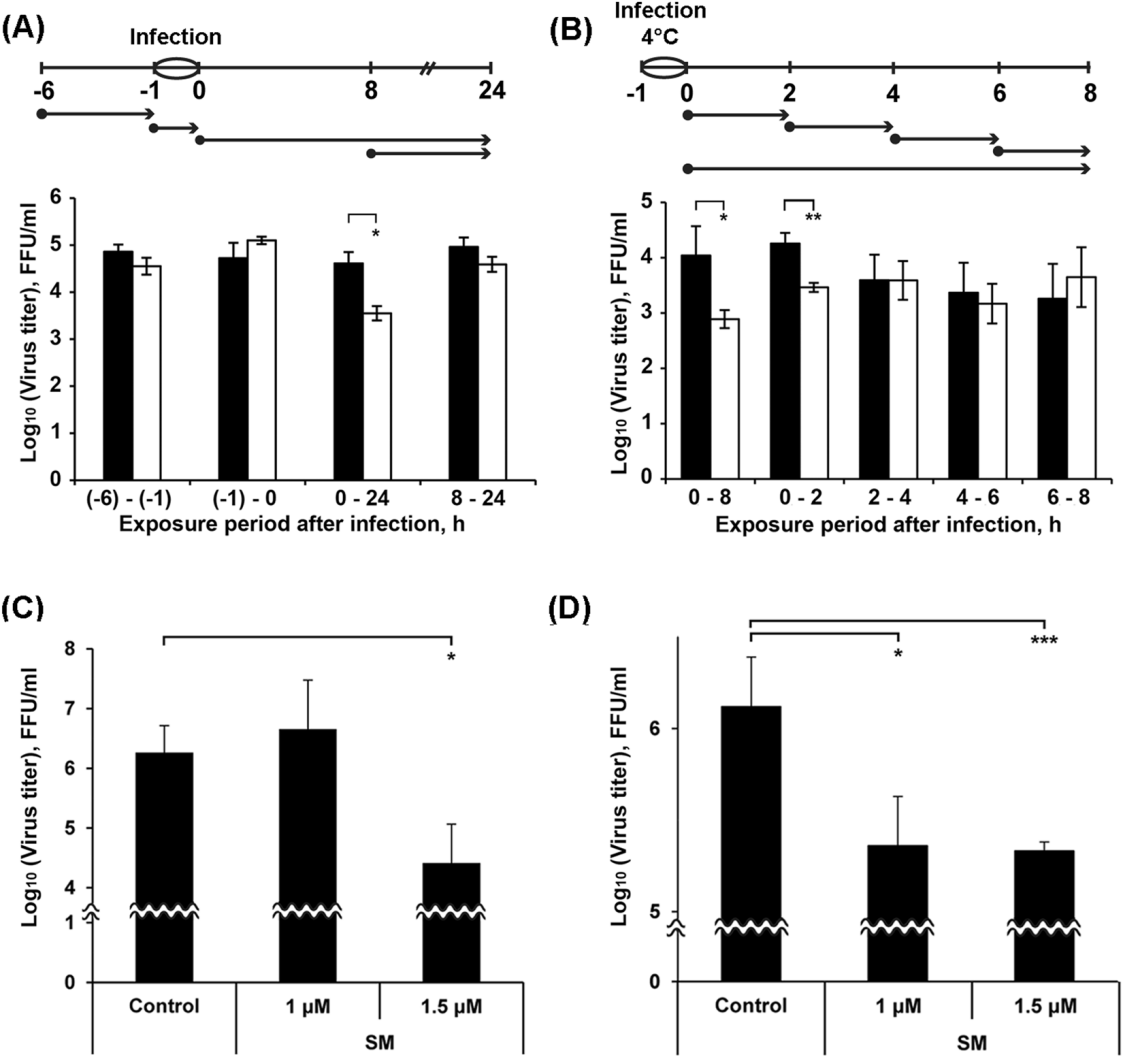
SM inhibits early steps of IVA infection. (**A**) and (**B**) Influence of the time of addition of SM on the virus production in cells. MDCK cells were infected with IVA at MOI 0.01 (A) or 0.001 (B) for 1 h (from -1 to 0 h). SM (1 µM) was added to cells at the indicate time points (•). In (**B**) after each incubation period (shown by arrows) the medium containing SM was replaced by the fresh infection medium. At the time points 24 h p.i. (**A**) and 8 h p.i. (**B**), the viral supernatants were collected and the viral yields were evaluated by FFA. Black columns – the mean viral yield for control cells; white columns – the mean viral yield for cells treated with SM. Data shown represent the mean ± SD of the results of three independent experiments. *p < 0.05, **p < 0.01. (**C**) SM inhibits binding of IVA to MDCK cells. MDCK cells were pre-chilled at 4 °C for 1 h and infected with IVA at MOI 0.01 followed by supplementation with SM (1 or 1.5 μM). After 3 h of incubation at 4 °C, cells were washed with PBS and overlaid with fresh medium. At 24 h p.i., viral supernatants were collected and the virus yields were determined by FFA. (**D**) SM inhibits penetration of IVA into MDCK cells. MDCK cells were pre-chilled at 4 °C for 1 h and incubated with virus (MOI 0.1) at 4 °C for 3 h. SM (1 or 1.5 μM) was then added to the cells and incubated for 60 minutes at 37 °C. Following inactivation and the neutralization of unpenetrated virus using acidic and alkaline PBS, respectively, cells were washed with PBS and overlaid with fresh medium. At 24 h p.i. viral supernatants were collected and the virus yields were determined by FFA. For (**C**) and (**D**): Control – untreated infected MDCK cells. Error bars represent the standard deviation of three independent experiments performed in triplicate. P-value: *<0.05; ***<0.001.

Using different SM treatment protocols and the synchronization of cells at infection, we attempted to elucidate the exact phase of the viral reproductive cycle affected by SM. The different treatment protocols are depicted in Fig. 3A and B. Arrows shows the time at which cells were incubated with SM: before, during or after infection. It has been reported that the time from entry into the cell to the production of new virus particles is on average 6–8 h, depending on the cell type^30,31^. First, the antiviral potentials of SM were tested at different time intervals (−6 to −1, −1 to 0, 0 to 24, and 8 to 24 h) relative to virus inoculation (Fig. 3A). Apparent SM-induced inhibition of virus amplification was observed only when SM was continuously present, starting with the post-infection period (Fig. 3A, time frame 0 to 24 h). SM did not exert its antiviral activity when it was added before infection (time frame −6 to −1 h) or during infection (time frame −1 to 0 h). It is noteworthy that no viral inhibition was observed when cells were treated during the second round of virus replication (Fig. 3A, time frame 8 to 24 h).

We investigated in detail the effects of SM in the time frame within one viral reproductive cycle (0 to 8 h) (Fig. 3B). Virus infection was synchronized by incubating cells with the virus at 4 °C (at this temperature, only virus attachment occurs) followed by incubation of cell culture at 37 °C to activate virus penetration. Infected cells were incubated with SM for the time periods 0 to 2, 2 to 4, 4 to 6, 6 to 8 and 0 to 8 h. Apparent SM-induced inhibition of virus amplification was observed when cells were incubated with SM from 0 to 8 and 0 to 2 h (Fig. 3B). Since SM treatment during the time period 2 to 8 h p.i. did not inhibit viral replication, we concluded that the anti-influenza activity of SM is exerted during the first two hours after infection. This time frame is known to cover the early steps of the influenza virus lifecycle, such as attachment, internalization and fusion^32^. Thus, inhibitory effect of SM is executed during the earliest steps of IVA replication cycle – virus binding to and uptake into host cells.

The ability of SM to inhibit viral attachment was tested using a virus binding assay. As shown in Fig. 3C, SM at 1 µM did not inhibit the attachment of viral particles to the cell surface. SM demonstrated viral inhibition only at 1.5 µM. SM also inhibited virus penetration into cells at 1 and 1.5 µM with similar efficiency, showing that a high concentration of SM is not required to achieve inhibition (Fig. 3D). In both cases, SM reduced the viral titer more than ten-fold.

Obtained results revealed that SM is probably able to inhibit HA activity although we didn’t observed the suppression of hemagglutination when SM was added to erythrocytes together with IVA. We supposed that SM binds to HA and therefore decreases its affinity to sialic acid, but efficiency of SM - HA interaction is not sufficiently high. In order to better understand how the interactions between SM and HA could occur molecular docking analysis was carried out.

**Figure 4:**
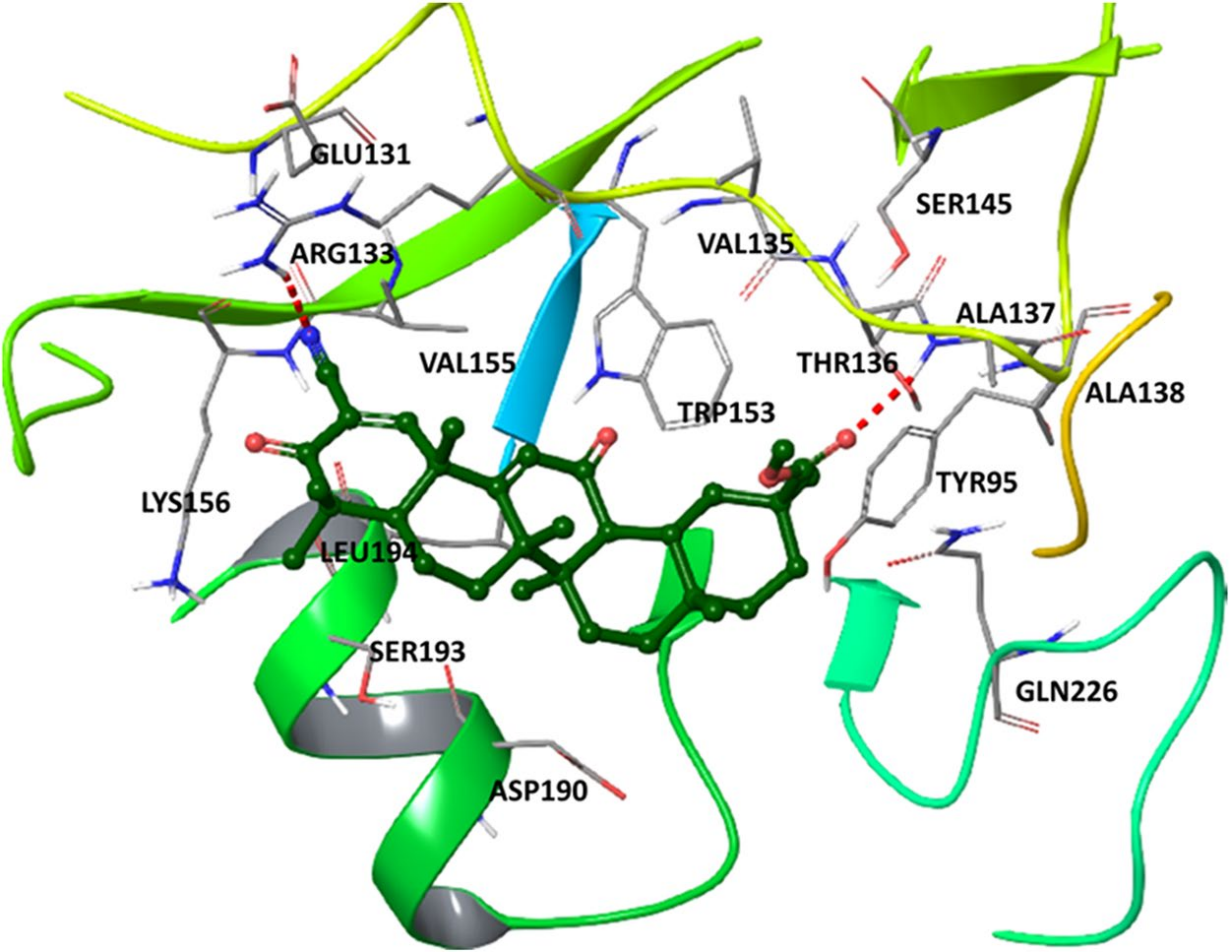
The docking complex of SM and hemagglutinin (PDB ID: 1RVT). SM is rendered as a stick and ball representation. Only residues situated less than 4 Å from the ligand has been shown. Hydrogen bonds between HA and SM are labeled as red.

Evaluation of binding poses of SM (Fig. 4) showed that its ester moiety has strong hydrogen bond with Ala137 of HA whereas nitrile formed hydrogen bond with Arg133 of the protein. Both residues are located in region conserved for binding of sialic acid^33^. Hydrophobic interactions between SM and HA typical for crystal complex of HA with HA’s receptor analog sialyllactose (LSTc) are also observed (Tyr95, Val135, Thr136, Val155, Asp190, Ser193 and Leu 194)^33^. Such results bear evidence that under some conditions SM in principle is able to bind to HA, but further direct experiments to prove it are needed.

**Figure 5:**
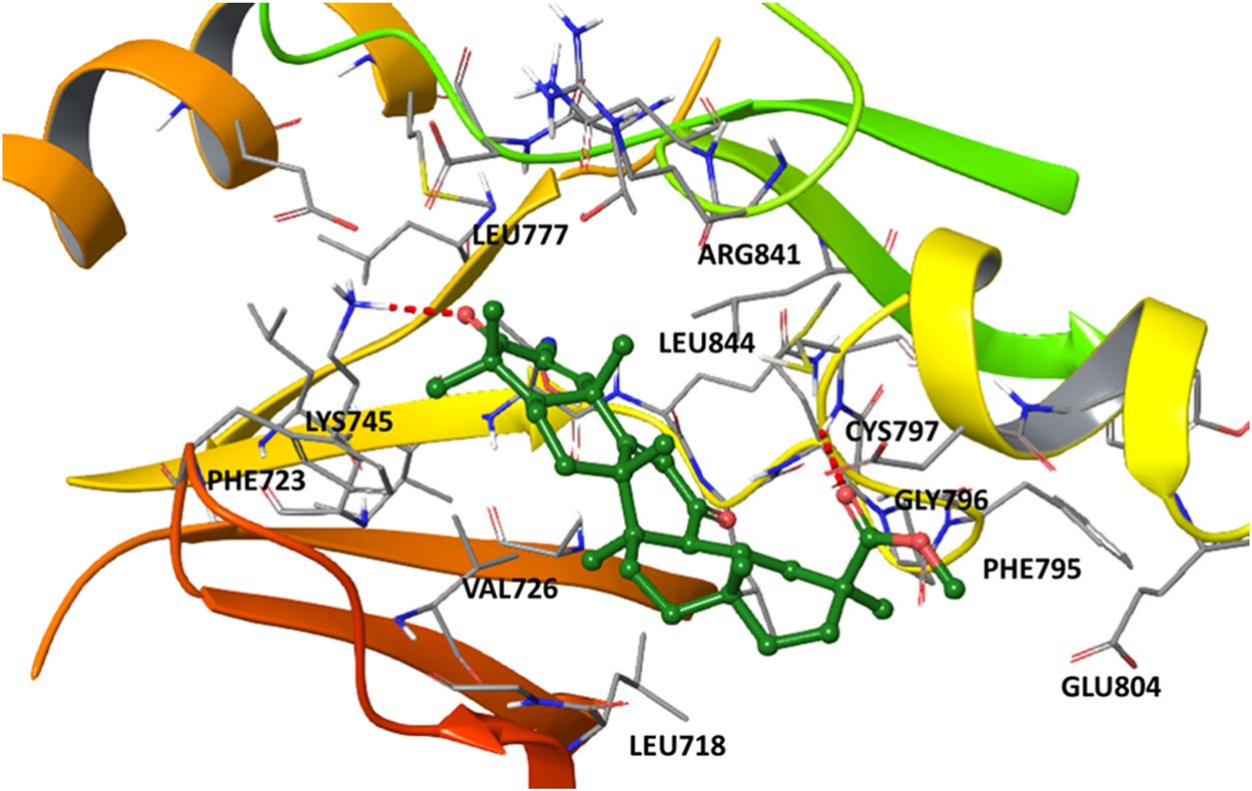
The docking complex of SM and EGFR (PDB ID: 1M17). SM is rendered as a stick and ball representation. Only residues situated less than 4 Å from the ligand has been shown. Hydrogen bonds between EGFR and SM are labeled as red.

EGFR as an indirect viral receptor to form a lipid raft-based signaling platform required for efficient IAV uptake^34^, so we examined whether SM is a binder of EGFR. SM docking pose within the EGFR binding site (Fig. 5) showed that ester moiety has hydrogen bond with Cys797 whereas ketone forms strong hydrogen bond with Lys745 indicated in literature as crucial for tyrosine kinase activity of the receptor^35^. Moreover SM possesses a lot hydrophobic interactions e.g. with Leu718, Phe723, Val726, Leu777, Gly796, Glu804, Arg841 as well as with Leu844. The same hydrophobic interactions (and H-bonds as well) were detected for another active compounds^35^ which confirms inhibitory potential of SM.

### SM inhibits IVA infection *in vivo*

**Figure 6:**
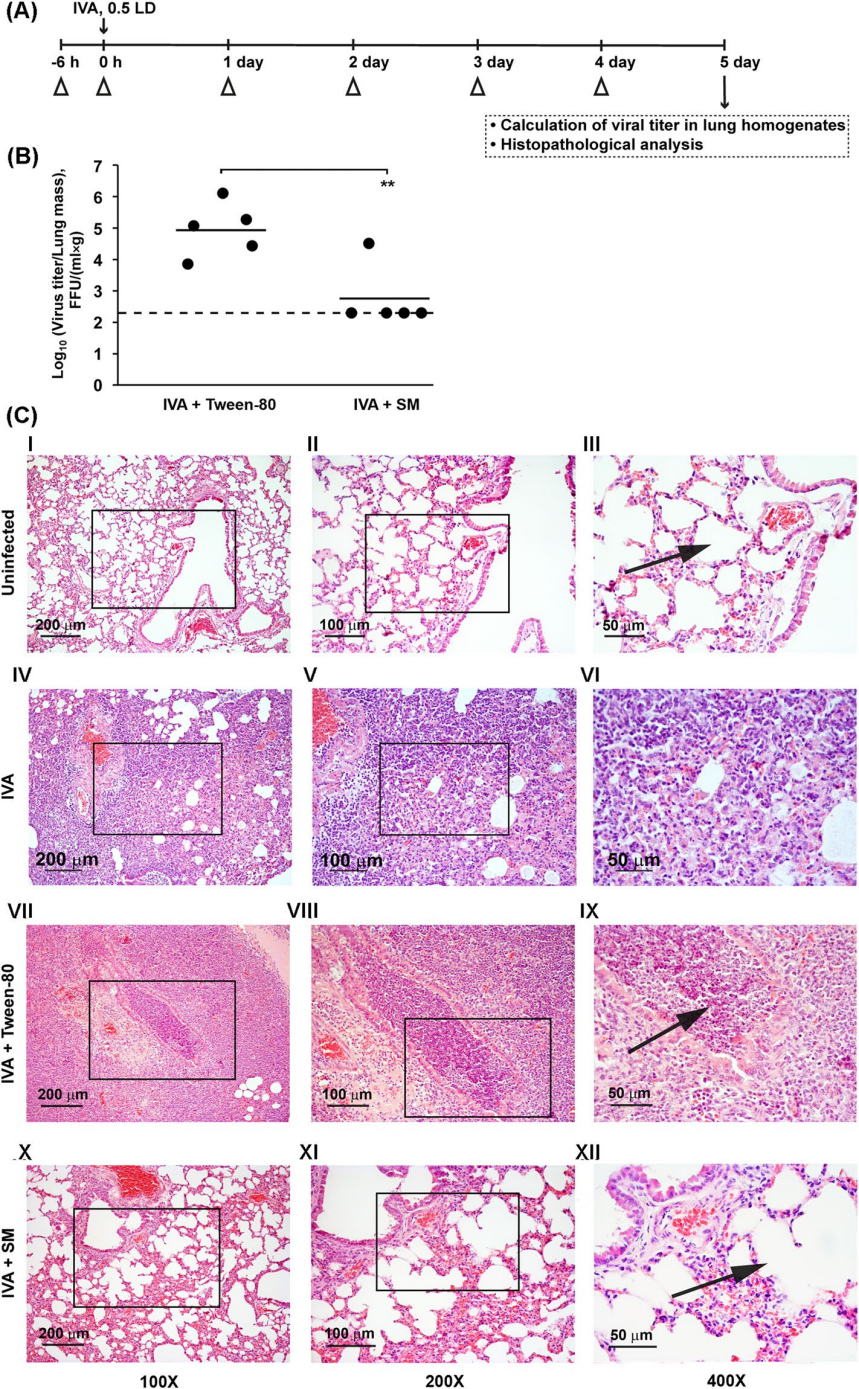
Intranasal treatment with SM prevents the development of non-lethal IVA infection in mice. (**A**) Scheme of the experiment. BALB/c mice were infected with IVA (0.5 LD) at 0 h, marked by an arrow. Infected mice were intranasally treated with vehicle (10% Tween-80) or 5 mg/kg of SM at the indicated time points (∆). On day 5 after infection, mice were weighed and euthanized and lung tissues were collected. **(B)** Virus titers in the lungs of mice that received intranasal treatment with SM on day 5 after IVA infection. Mean virus titers are shown by the horizontal bars. Dotted line represents the limit of sensitivity of the assay. The difference between SM-treated and Tween treated groups is statistically significant (**p < 0.01). **(C)** The effect of SM on the lung tissue of IVA-infected mice. Representative histological images of lung tissue of uninfected animals (I-III), IVA-infected animals (IV–VI), IVA-infected animals intranasally treated with 10% Tween-80 only (VII-IX) or with SM in 10% Tween-80 (5 mg/kg) (X–XII). Histopathological analysis revealed that untreated infected mice had severe diffuse pneumonia and severe bronchiolitis/peribronchiolitis, characterized by neutrophil-dominant inflammatory cellular infiltration (VI and IX, arrow). The intranasal administration of SM led to the disappearance of severe inflammatory changes in the lung tissue of infected mice (XII, arrow). Hematoxylin and eosin staining. Original magnification: I, IV, VII, X 100×; II, V, VIII, XI 200×; III, VI, IX, XII 400×. Black boxes show areas that were examined further at a higher magnification.

To evaluate the efficacy of SM against IVA *in vivo*, Balb/c mice were intranasally infected with 600 FFU of IVA. SM was administered intranasally at dose 5 mg/kg starting 6 h before infection, during infection and then once a day (Fig. 6A). The animals treated with the formulation only (10% Tween-80 in PBS) according to the same scheme as SM were used as a control group. SM treatment was shown to cause no weight loss in animals in comparison with control, thus indicating that the triterpenoid is non-toxic. Moreover, we recently showed that SM is non-toxic in a mouse model at doses up to 100 mg/kg (toxicity was evaluated by effect of SM onto the liver and kidney tissue (Supplementary Fig. S5). Also, we showed that 10% Tween-80 has not damage effect on mice lungs (Supplementary Fig. S6). On day 5 p.i., all animals were euthanized; post-mortem examinations were performed and lung viral titers were evaluated by FFA. As shown in Fig. 6B, treatment with SM effectively reduced viral replication in mice. The mean value of virus titers in the lungs of mice in the control group was 10^4,94^ FFU/(ml × g), whereas in four of five mice treated with SM, virus titer was near the limit of detection.

Pathomorphological examination of the lungs of mice from the IVA infected group and IVA infected group treated with formulation only (IVA+ Tween-80) revealed compelling morphological evidence of inflammatory tissue damage associated with severe viral pneumonia. The lungs of the control animals were edematous and exhibited multiple areas of diffuse and local hemorrhages on the pulmonary surface, consistent with the microscopic inflammatory response. Histopathological analysis revealed that the infected mice in these groups had severe inflammatory, circulatory and destructive changes in the lungs (Fig. 6C, IV-VI and VII-IX). The pathological changes in the lung tissue displayed a similar histopathological pattern with severe diffuse pneumonia with alveolar damage, characterized by interstitial neutrophil-dominant inflammatory cellular infiltration with the formation of foci of abscesses into the alveolar space, interstitial and alveolar edema and hemorrhage. Severe interstitial edema and neutrophil-dominant inflammatory cellular infiltration could be seen around small blood vessels and bronchioles as well as diffuse pneumonia with inflammatory cellular infiltrate and erythrocytes in the alveolar lumen. Alveoli were flooded with edema fluid mixed with fibrin, erythrocytes and inflammatory cells. Severe bronchiolitis/peribronchiolitis were also observed. Parts of the bronchial epithelium were necrotized and sloughed, and neutrophils and fibrin had exudated into the lumen of the bronchi. The bronchioles walls were infiltrated with neutrophils.

The intranasal administration of SM resulted in a significant reduction in the severity and in some cases an abrogation of gross and histopathological lesions in the lungs on day 5 p.i. The gross pathology was consistent with the microscopic evaluation. Four of the five animals in the SM-treated group demonstrated no evidence of infection by gross anatomic examination. SM treatment before and after infection led to reduction of inflammatory changes in the lung tissue of IVA-infected mice (Fig. 6C, X-XII). Histological analysis showed only slight interstitial edema and the slight interstitial inflammatory cellular infiltration with an absence of severe alveolar damage and bronchiolitis/peribronchiolitis in the lung tissue of IVA-infected animals treated with SM.

Thus, the intranasal treatment of IVA-infected mice with SM before and after infection resulted in significant suppression of lung pathology after influenza virus infection.

### SM inhibits influenza-induced production of TNF-α and IL-6 and improves factors of nonspecific resistance

**Figure 7:**
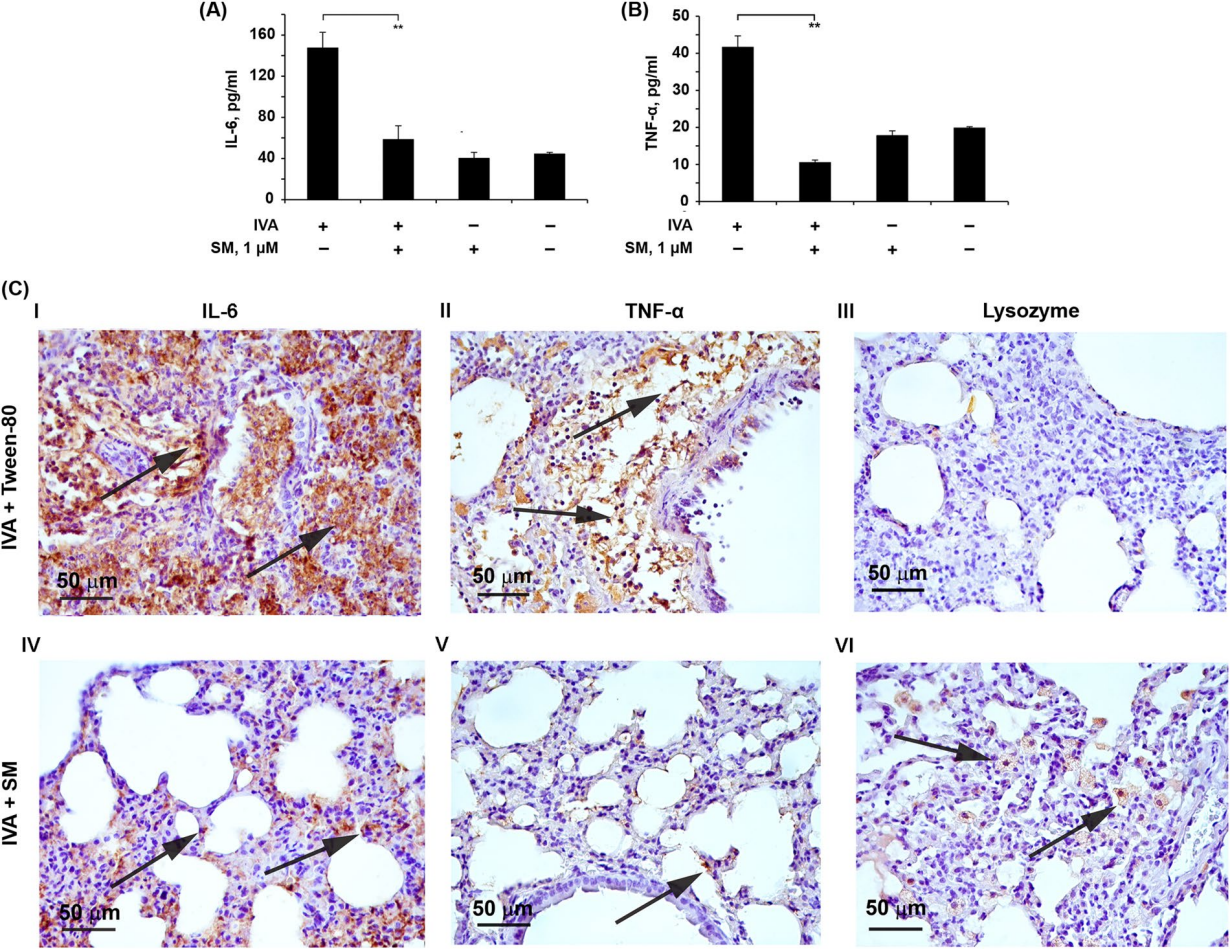
SM decreases pro-inflammatory cytokine production and improve factors of nonspecific resistance. A549 cells were infected with IVA at MOI 0.1 After 1 h of adsorption at 37 °C, the monolayers were washed twice with PBS and incubated with 1 µM of SM for 24 h. The levels of IL-6 **(A)** and TNF-α **(B)** in the supernatants were evaluated by ELISA. Data are represented as the mean ± SD. p-value: **p < 0.01. **(C)** Immunohistochemical staining of IL-6, TNF-alpha and Lysozyme positive cells in the lung tissue of IVA-infected mice treated with vehicle (10% Tween-80) (I, II, III) or 5 mg/kg of SM on day 5 p.i. (IV, V, VI). Arrows show IL-6 (I, IV), TNF-alpha (II, VII) and Lysozyme (III, VIII) positive cells in the lung tissue. Immunohistochemical staining of paraffin sections by anti-IL-6, anti-TNF-alpha and anti-Lysozyme monoclonal antibodies. Magnification 400×.

It is known that the inhibition of virus-induced cytokine release is also important for the treatment of influenza, so the influence of SM on the production of cytokines that have been closely linked to the progression of H1N1 infections was studied. We focused on TNF-α, which is a key cytokine in the cytokine storm associated with influenza pathogenesis and is likely to account for the escalation in severity^36^ and IL-6, the main pro-inflammatory cytokine released by the host during viral infection, which recent reports suggest is also responsible for many of the symptoms associated with influenza A infection^37^. A549 cells were infected with IVA at MOI 0.1. SM treatment was performed at 1 µM. Cytokine expression was detected 24 h p.i. by ELISA. We found that cells infected in this manner produced consistently high levels of TNF-α and IL-6. Both mediators were readily detected at 24 h p.i. SM at 1 µM did not affect cytokine expression of non-infected cells but inhibited the IVA-activated expression of IL-6 and TNF-α by 2.5- and 1.9-fold, respectively, almost to the basal levels (Fig. 7A and B).

We also performed the immunohistochemical (IHC) staining with anti-Lysozyme, anti-IL-6 and anti-TNF-α monoclonal antibodies of the lung tissue sections of IVA-infected mice (correspond to day 5 p.i.).

Morphometric analysis of IL-6, TNF-α and lysozyme immunohistochemical images demonstrated that the lung tissue of IVA mice treated with Tween-80 has a low expression of lysozyme and high expression of IL-6 and TNF-α (Fig. 7C, I, II and III). The numerical density of lysozyme positive cells was 1.5 ± 0.4, the volume density of TNF-α and IL-6 positive cells were 26.2 ± 1.8% and 44 ± 2.8%, respectively (Supplementary Table S2). The intranasal administration of SM led to increase of expression of lysozyme and to decrease of expression of IL-6 and TNF-α (Fig. 7C, IV, V and VI). 6.1-fold increase of the number of lysozyme positive cells (9.2 ± 0.7), 2.2-fold decrease of the volume density of TNF-α positive cells (12 ± 0.2%) and 2.4-fold decrease of the volume density of IL-6 positive cells was observed (Supplementary Table S2).

## Discussion

Licorice is one of the most widely used medicinal plants, and has been found in traditional formulas since antiquity. It is used to fight various symptoms associated with viral infections of the respiratory tract, as well as symptoms of hepatitis^38,39^. For the past 25 years, licorice-containing traditional herbal formulas have been investigated scientifically.

Recently, we demonstrated that SM exhibits anti-tumor, anti-oxidative and anti-inflammatory activities *in* *vitro* ^18–20,40^, and causes tumor cell death by inducing cell cycle arrest and apoptosis^18^. This study has revealed that SM also possesses anti-influenza activity. This activity was observed both *in vitro* and *in vivo*. *In vitro*, SM was shown to inhibit virus replication in both MDCK and A549 cells in a dose-dependent manner (Fig. 2B and Supplementary Fig. S1). Obtained data agree with the observed reduction in amount of NP and M2 proteins in SM-treated IVA-infected cells in comparison with control (IVA-infected cells) (Fig. 2C and Supplementary Fig. S2).

To understand the mechanisms mediating the antiviral effects of SM, a time-of-addition assay was performed. A significant inhibition in virus replication (up to 10 times) was observed only when SM was added to cells during the initial stage of viral infection (Fig. 3B, time frame 0 to 2 h), but pre-incubation of the cells with SM or incubation with SM during infection step did not affect IVA infection (Fig. 3A). SM did not exert antiviral activity when it was added 2 h p.i., which indicates that SM is ineffective at the stage of transcription and replication of the IVA genome and during the late stages of virus life cycle, i.e., during the assembly and release phases.

We questioned if the antiviral effect of SM is caused by its direct effect on the major IVA surface proteins HA and NA. Simultaneous incubation of virus inoculum with SM in both cell-free (virucidal activity assay) (Supplementary Fig. S7) and cell culture (time-of-addition assay, time frame −1 to 0 h) systems was found to not affect on subsequent IVA reproduction that clearly showed absence of SM’s direct virucidal action. The hemagglutination assay clearly showed that SM did not decrease the hemagglutination activity of IVA and therefore is not able to inhibit the receptor-binding activity of influenza HA (Supplementary Fig. S3). As well, we demonstrated that SM does not inhibit influenza NA activity (Supplementary Fig. S4). Similar results on NA activity inhibition were obtained earlier for wide range of pentacyclic triterpenoids^41^. Interestingly, despite lacking HA and NA inhibitory activity, SM had significant inhibitory effects on the binding and penetration of influenza virus to cells (Fig. 3C and D); the effect at binding stage was more pronounced than at the penetration stage. Earlier, a number of oleanane-type triterpenoids were shown to bind non-specifically to the influenza HA protein due to the hydrophobicity of the triterpenoid scaffold^33,42^. Performed molecular docking analysis revealed that binding pose of SM in the active site of HA is very similar to binding modes of already published ligand-HA complexes e.g. with LSTc^33^ or 2-deoxy-2,3-dehydro-N-acetyl-neuramic acid^43^ through interactions with Tyr95, Val135, Thr136, Ala137, Gln226, etc. Therefore, we suppose that the observed effect of SM is associated with its ability to bind to HA and reduce the effectiveness of HA binding to sialic acids, but the level of this effect is not sufficient to inhibit the hemagglutination activity of IVA.

Process of IVA penetration is known to be mostly depended on a range of the host cell factors, including some RTKs^44,45^ and their downstream effectors, precisely phospholipase C^46^, PI3K^47–49^, Akt^50,51^ and mTOR^52^. Since a lot of published works have shown that pentacyclic triterpenoids, being multitarget compounds, can effectively inhibit activity of all aforementioned proteins in various models^53^, we supposed that SM mediated suppression of IVA uptake is caused by its inhibitory effect on RTKs/PI3K/Akt/mTOR signaling axis. Considering the fact that a range of pentacyclic triterpenoids, including CDDO-Im, a structural analog of SM, effectively inhibit EGFR activity in different cell lines *in vitro* ^53^ and that some of them could directly bind to kinase domain of the receptor^54–56^, we examined whether SM is a binder of EGFR. Evaluation of binding poses of SM (Fig. 5) showed that ester moiety of SM forms hydrogen bond with Cys797 whereas ketone forms strong hydrogen bond with Lys745 indicated in literature as crucial for tyrosine kinase activity of the receptor^35^. Moreover, SM is able to form a net of hydrophobic interactions with Leu718, Phe723, Val726, Leu777, Gly796, Glu804, Arg841 as well as with Leu844. The same hydrophobic interactions (and H-bonds as well) were detected for a range of therapeutic inhibitors of EGFR (erlotinib, AEE788, staurosporine)^35^ which confirms inhibitory potential of SM (Fig. 5).

Cell membrane is a major player in IVA attachment and uptake^57^. In several studies it was shown that the inhibition of virus uptake by GLZ is the result of the suppression of the fluidity of the plasma membrane and viral envelope into which GLZ was incorporated^26,58^. The influence on the membrane fluidity was found for other triterpenoids too^59,60^. Moreover, triterpenoids due to similarity of their scaffolds with the structure of cholesterol are able to localize predominantly on the cell membrane, decrease the cholesterol content^61^, and induce the segregation of cholesterol within the lipid bilayer^62^. Proceeding from the aforesaid, the observed SM anti-IVA effects may result from their influence on the fluidity and cholesterol content of the cell membrane.

A mouse study was performed to determine the *in vivo* potential of SM against IVA. IVA-infected mice that did not receive treatment showed high lung viral titers on day 5 p.i. SM administration (5 mg/kg/day; i.n.) significantly reduced the levels of lung viral titers compared to those of untreated mice, and in four out of five mice, the value of virus titer was at the limit of sensitivity of the assay (in our case limit of detection was 2.3 log_10_) (Fig. 6B). In average log_10_ (viral titer) was reduced by 2 units.

Intranasal administration of SM before and after infection prevented the development of severe inflammatory changes in the lung tissue caused by the virus (Fig. 6C).

The involvement of NO and ROS overproduction and the synthesis of inflammatory mediators in the pathogenesis of influenza has been recognized as a major issue^63,64^. ROS produced during IVA infection is able to interact with NO, which leads to the formation of peroxynitrite (ONOO^−^) – the major mediator of oxidative stress-dependent injury in lung tissue^65^. A second mechanism that underpins acute lung injury and dysfunction of the respiratory system due to influenza infection is the overexpression of pro-inflammatory cytokines and chemokines (cytokine storm)^66^. We suppose that the observed anti-inflammatory activity of SM in mouse model of influenza-associated pneumonia may be linked with its high NO inhibitory and antioxidant activities, shown earlier for SM *in vitro* and hypoxia-induced lipid peroxidation in mice^40^.

It is known that the development of diseases accompanied with severe inflammation in the internal organs is associated with the reduction of the factors of nonspecific resistance (lysozyme, cathepsins), type I interferons and the growth of the pro-inflammatory cytokines (TNF-α, IL-1α, IL-6) in the tissues (cytokine storm), where inflammation develops. In this case more serious pathological changes are observed, such as diffuse alveolar damage, hyaline membrane formation, fibrin exudates and fibrotic healing. These are signs of severe capillary damage, immunopathological injury and persistent organ dysfunction^67^. Moreover, severe inflammatory cytokines/chemokines can spill over into the circulation and result in a systemic cytokine storm, which is responsible for multi-organ dysfunction^37,68,69^. We found that SM significantly suppressed the production of IL-6 and TNF-α *in vitro* (Fig. 7A,B), key cytokines in the pathogenesis of influenza infection^68,69^. Moreover, SM treatment of IVA mice significantly suppress lung pathology, improve the factors of nonspecific resistance and reduce the level of pro-inflammatory cytokines in the lung tissue (Fig. 7C, IV, V and VI; Supplementary Table S2). These effects explain our observations of a decreased local inflammatory response and attenuated infiltration of neutrophils in IVA infected mice receiving SM treatment.

In conclusion, the data presented here clearly demonstrate that SM exhibits anti-viral effects against influenza virus infection, leading to (i) the suppression of IVA production caused impaired virus binding to and uptake into cells; (ii) a decrease in virus titers in cultured cells and the lung tissue of infected mice; (iii) a reduction in lung lesions and attenuation of the cytokine cascade after viral infection. Thus, SM can be applied for the inhibition of virus replication and also for the prevention of severe complications in the lungs caused both by the virus itself and the cytokine storm and resulting tissue hypoxia.

## Materials and Methods

### Reagent and compounds

The chemical synthesis of the glycyrrhetinic acid derivative Soloxolone methyl (SM) has been described before^18^. This compound has been fully characterized by chemical analysis and nuclear magnetic resonance. Glycyrrhizin was obtained from a licorice extract^16^. SM and glycyrrhizin were dissolved in DMSO (10 mM), and stock solutions were stored at −20 °C.

### Virus and cells

Madin-Darby canine kidney cells (MDCK) and human lung carcinoma epithelial cells (A549) were obtained from the Russian Cell Culture Collection (St. Petersburg, Russia) and were cultured in Dulbecco’s modified Eagle’s medium (DMEM) supplemented with 10% (v/v) heat-inactivated fetal bovine serum (FBS) and antibiotic-antimycotic solution (100 U/ml penicillin, 100 μg/ml streptomycin and 0.25 μg/ml amphotericin). Cells were incubated at 37 °C in a humidified atmosphere containing 5% CO_2_/95% air (hereafter referred to as standard conditions). The influenza A/WSN/33 (H1N1) virus was kindly provided by Dr. Elena Goncharova (Institute of chemical biology and fundamental medicine SB RAS, Novosibirsk, Russia); it was amplified and titrated in MDCK cells and stored at −80 °C until use. The titer of virus stocks was determined by the focus forming assay described below. The cells were seeded in cell culture plates in Iscove’s modified Dulbecco’s medium (IMDM), supplemented with 10% (v/v) FBS and antibiotic-antimycotic solution and cultivated to a confluent monolayer. The medium used for virus infections (infection medium) consisted of IMDM, supplemented with antibiotic-antimycotic solution and 2 μg/ml N-tosyl-L-phenylalanine chloromethyl ketone (TPCK)-treated trypsin.

### Cell viability analysis

MDCK and A549 cells were seeded in 96-well plates at 1 × 10^4^ cells/well. The plates were incubated under standard conditions for 48 h. The medium was replaced with fresh medium containing serially diluted SM and the cells were further incubated for 24 h under standard conditions. Aliquots of [3-(4,5-dimethylthiazol-2-yl)-2,5-diphenyltetrazolium bromide] (MTT) solution (10 μl, 5 mg/ml) were added to each well, and the incubation was continued for an additional 3 h. The dark blue formazan crystals that formed within live cells were solubilized with DMSO, and the absorbance was measured at 570 nm in a Multiscan RC plate reader (Thermo LabSystems, Finland). The IC_50_ was determined as the compound concentration required to decrease the A_570_ to 50% of the control (DMSO, no compound), and was determined by interpolation from the dose-response curves.

### Focus forming assay (FFA)

Viral titers were determined by the focus forming assay in MDCK cells^70^. Cell culture supernatants were transferred to 96-well plates containing confluent MDCK cells. The supernatants were serially diluted ten-fold with infection medium and incubated with MDCK cells for 24 h under standard conditions. Thereafter, MDCK monolayers were subsequently washed twice with PBS and fixed with ice-cold 80% acetone for 15 min at room temperature. Viral foci were stained using a mouse monoclonal antibody against influenza A NP (Millipore, USA), goat anti-mouse IgG conjugated with biotin (Sigma-Aldrich, USA), peroxidase labeled streptavidin (MP Biomedicals, USA) and 3-amino-9-ethylcarbazole (AEC) (Sigma-Aldrich, USA). Focus-forming units (FFU) (NP-positive red-colored cells located apart from another one at a distance of two uncolored cells) were then calculated and viral titers were expressed as FFU per ml.

### Virus yield reduction assay

MDCK or A549 cells were grown to confluence in 24-well plates. Cells were infected with IVA (MOI = 0.01 FFU/cell) either with or without 1 µM of SM at 37 °C under standard conditions for 1 h. The residual inoculum was discarded, cells were washed twice with PBS and overlaid with fresh infection medium with or without 1 µM SM. The cultures were incubated under standard conditions. At 24 h p.i., cells were subjected to one cycle of freeze (−20 °C)/thaw (20 °C) and then virus titers were determined by FFA.

### Indirect immunofluorescence assay (IFA)

A549 cells were seeded into four-chamber culture slides (BD Falcon, BD Biosciences, USA) (5 × 10^4^ cells per chamber). The slides were incubated under standard conditions for 24 h. Thereafter, the cells were infected or mock-infected with IVA at MOI = 0.1 FFU/cell for 1 h at 37 °C in 5% CO_2_. The residual inoculum was discarded; cells were washed with PBS and overlaid with fresh infection medium with or without 1 μM SM. The cultures were incubated at 37 °C in 5% CO_2_ for 24 h, fixed for 30 min with 4% paraformaldehyde. Permeabilization was done with 0.02% Triton-X100, followed by blocking with 1% BSA in PBS. The cells were incubated with mouse monoclonal antibodies against IVA nucleoprotein (NP) (Millipore, USA) or M2 protein (Abcam, USA) for 1 h at 37 °C. The cell nuclei were stained with 4′,6-diamidino-2-phenylindole (DAPI). Immunofluorescence was observed using an LSM 510 confocal laser scanning microscope (Zeiss, Germany).

### Hemagglutination (HA) assay

The hemagglutination assay was used to assess the effect of SM on virus adsorption^71^. Briefly, a two-fold serial dilution of SM (0.098–100 μM) in 0.25% sodium citrate in PBS was mixed with an equal volume of IVA suspension (25 μl/well, containing 4 HAU) for 1 h at 4 °C in round-bottom 96-well plates. Subsequently, 50 μl of 0.5% chicken erythrocytes in 0.25% sodium citrate in PBS were added to each well. After 40 min incubation at 4 °C, the hemagglutination reaction was observed.

### Neuraminidase (NA) inhibition activity

The NA activity was measured in a fluorescence-based assay using the NA-Fluor™ Influenza Neuraminidase Assay Kit (Applied Biosystems, USA) in accordance with the manufacturer’s protocol. Briefly, diluted virus was mixed with the SM (ten-fold serial dilutions from 10 µM to 0.01 nM) and incubated at 37 °C for 30 min. The substrate working solution was then added, and the mixture was incubated at 37 °C for 1 h. The reaction was stopped by adding Stop solution. The fluorescence intensity was measured using a CLARIOstar microplate reader (BMG Labtech GmbH, Germany) with excitation and emission wavelengths of 360 and 450 nm, respectively.

### Time-of-addition assay

(a) To estimate influence of pre-, during- and post-infection incubation of SM on IVA replication, MDCK cells were grown in 24-well plates to confluence and infected with IVA (MOI = 0.01 FFU/cell) for 1 h under standard conditions. SM was added at a concentration of 1 µM before, during or after IVA infection. For pre-infection, SM was added to cells and cells were incubated with SM for 5 h at 37 °C followed by washing three times with PBS before virus infection. For co-infection, the cells were simultaneously incubated with IVA and SM. After 1 h, the virus-drug mixture was removed (time zero), cells were washed three times with PBS and fresh infection medium was added. For post-infection, the cells were first infected with IVA for 1 h, washed three times with PBS, and then SM was added to the cells at the indicated time points (0, 2, 8 and 10 h post-infection [p.i]). At 24 h p.i., monolayers were subjected to one freeze/thaw cycle and the viral yields were determined by FFA. (b) To determine the steps of the IVA life cycle sensitive to SM treatment, confluent monolayers of MDCK cells in 24-well culture plates were inoculated with IVA (MOI = 0.001 FFU/cell) for 1 h at 4 °C to ensure the viruses would replicate synchronously. The residual inoculum was discarded, cells were washed three times with PBS to remove unbound virus and incubated in a fresh portion of infection medium under standard conditions (time zero). The test medium containing SM at 1 μM was added at 0 to 2, 2 to 4, 4 to 6, 6 to 8 h or 0 to 8 h. After each incubation period, the monolayers were washed three times with PBS and incubated with fresh infection medium at 37 °C. At 8 h p.i. the monolayers were subjected to one freeze/thaw cycle and the viral yield was determined by FFA.

### Virus binding assay

The influence of SM on virus binding to the cell surface was studied according to a previously described method^72^. Briefly, confluent MDCK cells were cooled on ice for 1 h. Thereafter, IVA at MOI = 0.01 FFU/cell and SM at concentrations of 0.75, 1 or 1.5 μM were added simultaneously. Cells were held at 4 °C for a further 3 h to allow virus adsorption. Unabsorbed virus-containing supernatants were removed, the cells were washed twice with PBS and fresh infection medium was added. Following incubation under standard conditions for 24 h, cells were subjected to one freeze/thaw cycle and viral yield was determined by FFA.

### Penetration assay

The penetration assay developed by Albin *et al*.^73^ was used to estimate the ability of SM to inhibit IVA entry. Briefly, the MDCK cell monolayer was prechilled on ice for 1 h. The cell monolayer was infected with IVA at MOI = 0.1 FFU/cell and incubated for 3 h at 4 °C to allow virus adsorption. After adsorption, SM at concentration of 1 μM was added and cells were shifted to 37 °C for 1 h to allow the virus to penetrate into the cells. The infected cells were then treated with acidic PBS (pH 3) for 1 min to inactivate non-penetrated virus, and then PBS (pH 11) was immediately added to neutralize the buffer. The neutral PBS was removed and the cells were washed twice with normal PBS (pH 7.4) and overlaid with infection medium. The cells were then incubated under standard conditions for a further 24 h, subjected to one freeze/thaw cycle and viral yield was determined by FFA.

### In silico studies

The protein structure for docking studies, crystal complexes of HA with siallylactose (LTSc) and complex of EGFR with erlotinib were selected from the PDB (PDB ID: 1RVT, resolution 2.50 Å and PDB ID: 1M17, resolution 2.60 Å) and prepared in Protein Preparation Wizard [Protein Preparation Wizard; Epik, Schrödinger, LLC, New York, NY, 2016; Impact, Schrödinger, LLC, New York, NY, 2016; Prime, Schrödinger, LLC, New York, NY, 2016], under the default settings. Three dimensional structure, conformation and protonation states of SM were generated by LigPrep [LigPrep, Schrödinger, LLC, New York, NY, 2016] (at pH 7.4). Finally, Glide [Glide, Schrödinger, LLC, New York, NY, 2016] was used for docking at the SP level under default settings (sampling nitrogen inversion, sampling ring conformations with energy window equal to 2.5 kcal/mol, penalizing nonplanar conformation of amides up to 100 steps during energy minimization and performing postdocking optimization) of each SM conformer to protein model. Each pose was ranked according to affinity score, and the highest scored pose was chosen for further analysis.

### Animal studies

Inbred female Balb/c mice at the age of 6–8 weeks (16–20 g) were obtained from the State Research Center of Virology and Biotechnology VECTOR (Koltsovo, Russia). The mice were quarantined for 72 h prior to experimental manipulation, during which time the animals were observed for signs of disease and/or physical abnormalities. Animals were kept in the vivarium of the Institute of Chemical Biology and Fundamental Medicine, SB RAS, with a natural light regime on a standard diet for laboratory animals [GOST (State Standard) R 5025892] in compliance with the international recommendations of the European Convention for the Protection of vertebrate animals used for experimental studies (1997), as well as the rules of laboratory practice in the performance of pre-clinical studies in the Russian State Standards (R 51000.3–96 and 51000.4–96). The experimental protocols were approved by the Committee on the Ethics of Animal Experiments with the Institute of Cytology and Genetics of the Siberian Branch of the Russian Academy of Sciences.

Mice were anesthetized by intraperitoneal injection of tribromoethanol (Avertin®) at a dose of 250 mg/kg and then infected intranasally with 600 FFU of IVA (50% of LD_50_) in 50 µl of PBS. Soloxolone methyl was dissolved in 10% Tween-80 (Sigma-Aldrich, USA) in PBS and administered intranasally at dose 5 mg/kg in 50 µl 6 h before infection, during infection and 24, 48, 72 and 96 h after infection. Control animals were treated with the formulation only (10% Tween-80 in PBS). The mice were weighed and euthanized on day 5 after infection. The lung tissue of mice was collected for (a) preparation of homogenates for virus titer determination by FFA and (b) histopathological analysis.

### Histopathological analysis

For histological evaluation the lungs were fixed in 10% neutral-buffered formalin, routinely processed, and embedded in paraffin. Paraffin sections (5 μm) were stained with hematoxylin and eosin, microscopically examined and scanned.

Lung sections for immunohistochemical (IHC) studies (3–4 μm) were deparaffinated, rehydrated; antigen retrieval was carried out in a microwave oven at 700 W. The samples were incubated with the IL-6 (Abcam, ab 7737), TNF-α (SONY, Mab11) and Lysozyme (Diagnostic Biosystem, RP028) first specific antibodies. Then the sections were incubated with HRP conjugates and second antibodies (Spring Bioscience detection system), exposed with DAB substrate, and stained with Mayer’s hematoxylin.

Images were obtained using a microscope Axiostar plus equipped with a digital camera Axiocam MRc5 (Zeiss, Germany) at 100×, 200× and 400× magnifications. Each experimental group contained 8–10 mice specimens, 10–15 randomly-selected microscopic fields were studied in each specimen.

Lung histopathological changes in the influenza infected mice included diffuse interstitial pneumonia, alveolar damage, inflammatory cellular infiltration, bronchiolitis/peribronchiolitis, interstitial and alveolar edema, and hemorrhage.

Stereological quantification of the lung samples after IHC staining was performed by point counting, using closed test-system at a magnification 400×. The test-system used had 100 testing points in a testing area equal to 3.2 × 10^6^ μm^2^. The volume density (Vv) of IL-6 and TNF-α positive cells representing the volume fraction of tissue occupied by this compartment was determined from the points lying over this structure and calculated using the following formula: Vv = (*P*_structure_/*P*_test_) × 100%, where *P*_structure_ denotes the number of points over the structure and *P*_test_ represents the total number of test points, 100 in this case. The numerical density (Nv) of Lysozyme positive cells indicating the number of particles in the unit tissue volume was evaluated as a number of particles in the square unit, 3.2 × 10^6^ μm^2^ in this case. The histological data were statistically processed using the two-tailed Student’s t-test (data are expressed as mean ± SD). Differences were considered statistically significant at p < 0.05.

### ELISA

Confluent monolayers of A549 cells were infected with IVA at MOI 0.1 FFU/cell at 37 °C for 1 h. Then cells were washed twice with PBS and SM was added at a concentration of 1 μM, followed by incubation for 24 h under standard conditions. Supernatants from mock- or IVA-infected cells were collected and analyzed for IL-6 and TNF-α by ELISA (JSC Vector-Best, Russia) according to the manufacturer’s protocol. Briefly, 100 µl of supernatant and 100 µl of Dilution Buffer were placed to wells with immobilized monoclonal antibodies against IL-6 or TNF-α and incubated for 2 h at 37 °C and 700 rpm in thermostatic shaker ST-3M (ELMI Ltd, Latvia). After that, wells were sequentially filled with 100 µl of biotinylated anti-IL-6 or anti-TNF-α antibody solution, horseradish peroxidase labeled streptavidin (HRP-streptavidin) solution, 3,3′,5,5′-tetramethylbenzidine (TMB) solution and incubated for 1 h at 37 °C (for biotinylated antibodies and HRP-streptavidin) or 30 min at room temperature (for TMB) on the shaker (700 rpm). Before each addition, wells were washed five times with Wash buffer. Stop solution was finally added to each well, and after 3 min of incubation at room temperature, the absorbance was measured at 450 nm using a Multiscan RC plate reader (Thermo LabSystems, Finland). The experiment was repeated twice in three parallel measurements.

### Statistical analysis

Data are expressed as the mean ± SD. Statistical analysis was performed using the two-tailed unpaired t-test. P-values of less than 0.05 were defined as statistically significant.

## Electronic supplementary material

Dataset 1
