# Supplementary material for: Soloxolone methyl inhibits influenza virus replication and reduces virus-induced lung inflammation
Source: Sci Rep. 2017 Oct 25;7:13968. doi: 10.1038/s41598-017-14029-0 (PMC5656677; doi:10.1038/s41598-017-14029-0)
Supplement: Supplementary file 1 — Dataset 1 [file 41598_2017_14029_MOESM1_ESM.doc]

***Soloxolone methyl inhibits influenza virus replication and reduces virus-induced lung inflammation***

Andrey V. Markov,^1^ Alexandra V. Sen’kova,^1^ Dawid Warszycki,^2^ Oksana V. Salomatina,^3^ Nariman F. Salakhutdinov,^3^ Marina A. Zenkova,^1^ Evgeniya B. Logashenko^1*^

*^1^Institute of Chemical Biology and Fundamental Medicine, Siberian Branch Russian Academy of Sciences, 8, Lavrent’ev ave., Novosibirsk, 630090, Russian Federation*

*^2^Institute of Pharmacology, Polish Academy of Sciences, 12, Smętna street, Kraków, 31-343, Poland*

*^2^N. N. Vorozhtsov Novosibirsk Institute of Organic Chemistry, Siberian Branch Russian Academy of Sciences, 9, Lavrentjev ave., Novosibirsk, 630090, Russian Federation*

**SUPPLEMENTARY MATERIALS**

**Fig. S1.** **Effect of SM on IVA titers after 24 h of incubation.** MDCK cells were infected with IVA at MOI 0.01. The production of infectious virus in the supernatants was examined by the FFA. Effect of SM on MDCK cell viability was measured by MTT assay and expressed as a percentage of viable SM-treated IVA-infected cells vs untreated IVA-infected control cells (100%). Error bars represent the standard deviation of three independent experiments. p-value: * < 0.05, ** <0.01, *** < 0.001.

| **Table S1**  **Anti-influenza activity of GLZ *in vitro*** | | | | | |
| --- | --- | --- | --- | --- | --- |
| **IVA strain** | **MOI** | **[GLZ], mM** | **T_GLZ treatment_, h** | **Fold change in IVA titers ^(a)^** | **Ref.** |
| A/WSN/33 (H1N1) | 0.01 | 1 | 24 | 0 | [our data] |
| A/Vietnam/1203/04 (H5N1) | 0.01 | 0.2 | 24 | 10 | [23] |
| A/Aichi/2/68 (H3N2) | 0.05 | 1 | 16 | 10 | [24] |
| ^(a)^ The fold change in IVA titers between untreated and GLZ-treated IVA-infected cells.  23. Michaelis, M. et al. Glycyrrhizin exerts antioxidative effects in H5N1 influenza A virus-infected cells and inhibits virus replication and pro-inflammatory gene expression. PLoS One 6, e19705, doi: 10.1371/journal.pone.0019705 (2011).  24. Wolkerstorfer, A., Kurz, H., Bachhofner, N. & Szolar, O. H. Glycyrrhizin inhibits influenza A virus uptake into the cell. Antiviral Res 83, 171-178, doi: 10.1016/j.antiviral.2009.04.012 (2009). | | | | | |


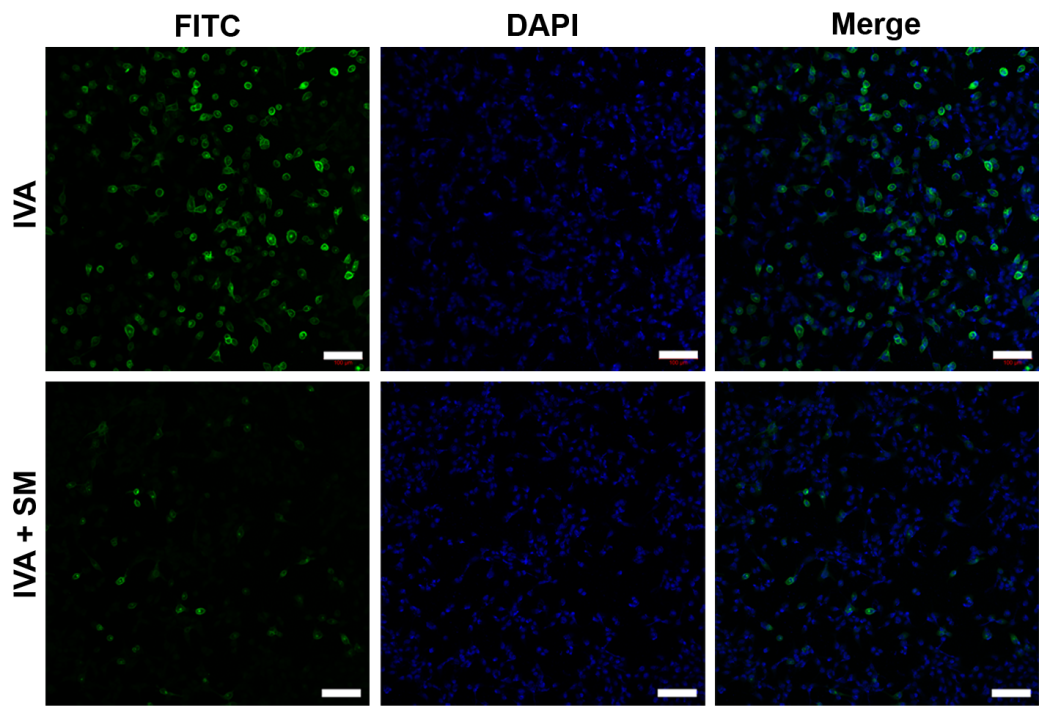


**Fig. S2.** **Effect of SM on the expression of influenza virus M2 protein in A549 cells.** Confluent A549 cells were infected with IVA (MOI = 0.1) and then treated with SM (1 µM). At 6 h p.i. the cells were fixed with paraformaldehyde and expression of viral M2 protein was detected by IFA using antibody sandwich complex (primary anti-M2 antibody / biotin-conjugated secondary antibody / FITC-conjugated streptavidin). Nuclei were counterstained with DAPI. Cells were viewed using laser scanning confocal microscope with a 10× objective. Bars, 100 µm.


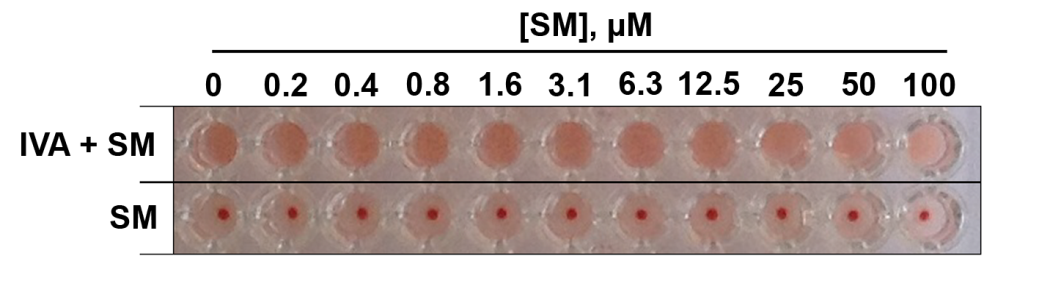


**Fig. S3.** **Effect of SM on IVA-induced hemagglutination.** Two-fold serial diluted SM was mixed with equal volume of IVA suspension (4 HAU) (IVA + SM) or 0.25% sodium citrate in PBS (SM). After 1 h incubation at 4°C, 0.5% chicken RBCs in 0.25% sodium citrate in PBS were added. Plate was incubated at 4°C for 40 min followed by hemagglutination reaction was observed.


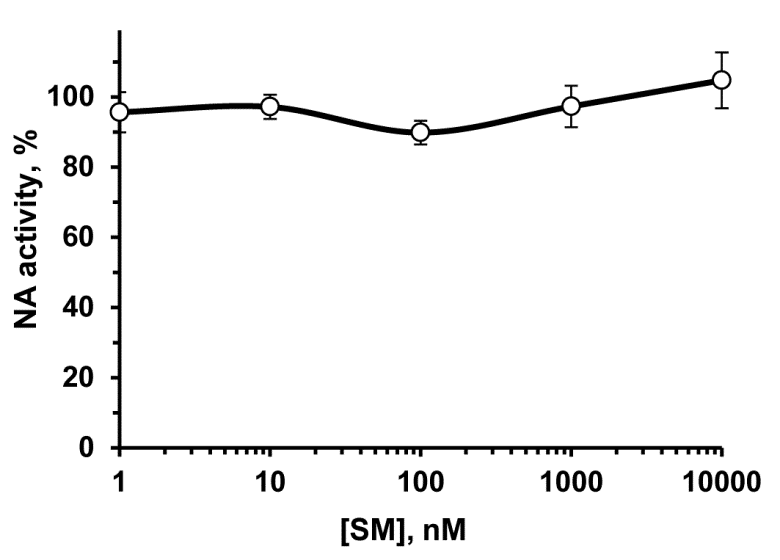


**Fig. S4.** **Effect of SM on influenza virus NA activity.** Diluted IVA (4.8×10^2^ FFU) was mixed with SM at mentioned concentrations and incubated at standard conditions for 30 min. The viral NA enzymatic activity was measured using NA-Fluor^TM^ Influenza Neuraminidase Assay Kit (Applied Biosystems, USA). Data represent levels of viral NA activity after SM treatment in comparison with NA activity of intact virus. Error bars depict the standart deviation from the mean of triplicate samples.


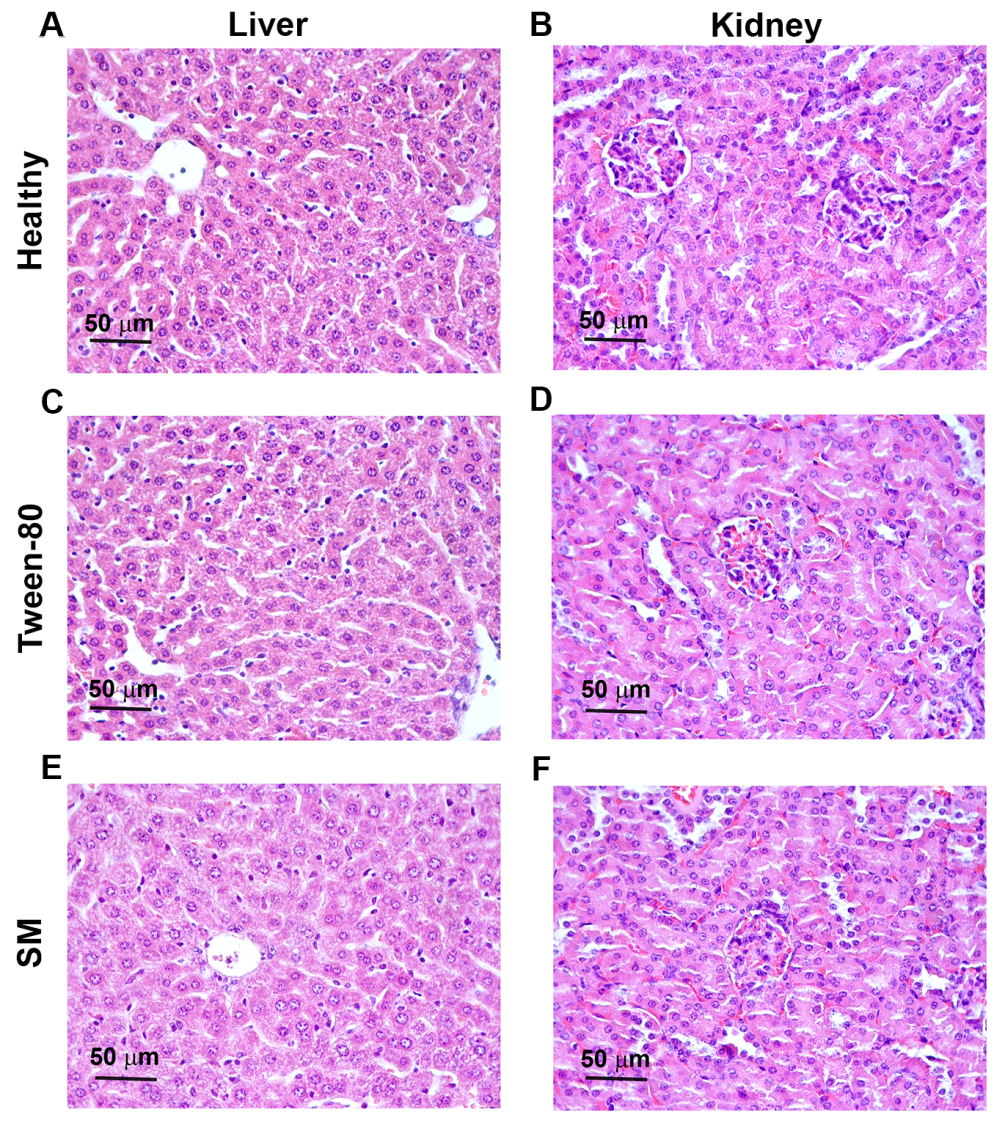


**Fig. S5. The effect of SM on the liver and kidney tissue.** Morphological structure of the liver and kidney tissue of healthy mice without treatment (A, B) and after intravenous administration of 10% Tween-80 (C, D) and SM at a dose 100 mg/kg (E, F). Histopathological analysis revealed that intravenous administration of 10%Tween-80 and SM at a dose 100 mg/kg did not led to increase in destructive changes in the liver and kidney tissue. Hematoxylin and eosin staining. Original magnification 400×.

As a rule, the liver tissue of healthy animals contains 4.7 ± 0.5% of hepatocytes with degeneration and 6.2 ± 0.7% of necrosis of liver parenchyma. Intravenous administration of Tween-80 and SM at a dose 100 mg/kg to healthy animals did not lead to increase in destructive changes in the liver tissue and did not aggravate the liver injury.

Kidneys of healthy animals have about 10% of epithelial cells of the proximal and distal tubules with destructive changes, which include more dystrophy and less necrosis. Intravenous administration of Tween-80 and SM at a dose 100 mg/kg to healthy animals did not increase destructive changes in the kidney tissue. The percentage of destructive changes in liver and kidney tissue was counted using closed test-system consisted of 100 testing points in a testing area equal to 3.2 × 10^6^ μm^2^.

**Fig. S6. The lung tissue of healthy (uninfected) mice after Tween-80 administration.** Representative histological images of lung tissue of uninfected animals intranasally treated with 10% Tween-80 only. Histopathological analysis revealed that uninfected mice had no pathological changes in the lung tissue after 10% Tween-80 intranasal administration. Hematoxylin and eosin staining. Original magnification: 100×, 200×, 400×. Black boxes show areas that were examined further at a higher magnification.

| **Table. S2. Morphometric analysis of immunohistochemical staining of lysozyme, TNF-α and IL-6 in the lung tissue of mice with influenza A/H1N1 (IVA mice)** | | |
| --- | --- | --- |
|  | **Tween-80** | **SM 5 mg/kg** |
| Lysozyme positive cells, Nv ^a^ | 1.5±0.4 | 9.2±0.7* |
| TNF-alpha positive cells, Vv, % ^b^ | 26.2±1.8 | 12±0.2* |
| IL-6 positive cells, Vv, % | 44±2.8% | 18±0.3%* |
| * statistically significant difference relative to Tween-80 group, p ≤ 0.05;  ^a^ the numerical density (Nv) indicating the number of particles in the unit tissue volume;  ^b^ the volume density (Vv) representing the volume fraction of tissue occupied by this compartment. | | |


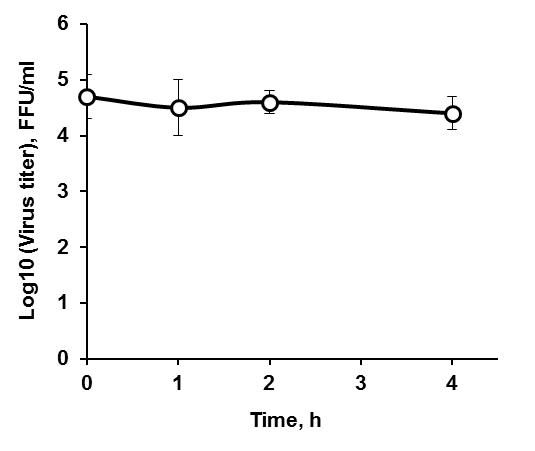


**Fig. S7. SM does not display virucidal activity against IVA.** IVA-containing suspension (100 µl; 10^4,7^ FFU/ml) was incubated with equal volume of 2 µM of SM for 0, 1, 2 and 4 h at 37°C under standard conditions. Residual virus titer after treatment was evaluated by FFA.
